# Zinc oxide enriched peat influence *Escherichia coli* infection related diarrhea, growth rates, serum and tissue zinc levels in Norwegian piglets around weaning: five case herd trials

**DOI:** 10.1186/s40813-017-0060-7

**Published:** 2017-06-28

**Authors:** M. Oropeza-Moe, C.A. Grøntvedt, C.J. Phythian, H. Sørum, A.K. Fauske, T. Framstad

**Affiliations:** 1 Department of Production Animal Clinical Sciences, Norwegian University of Life Sciences (NMBU) Faculty of Veterinary Medicine, Campus Sandnes, Sandnes, Norway; 2 Norwegian Veterinary Institute, Oslo, Norway; 3 Faculty of Veterinary Medicine, Department of Food Safety and Infection Biology, Norwegian University of Life Sciences, Oslo, Norway; 4 Department of Production Animal Clinical Sciences, Norwegian University of Life Sciences (NMBU) Faculty of Veterinary Medicine, Campus Adamstuen, Adamstuen, Norway

**Keywords:** Piglet, ZnO enriched peat, Coated ZnO, Diarrhea, Growth rates, Serum ZnO, One Health

## Abstract

**Background:**

Zinc oxide (ZnO), commonly used to control post-weaning diarrhea in piglets, has been highlighted as of potential concern from an environmental perspective. The aim of this field trial was to examine effects of different sources and levels of ZnO added to peat on average daily weight gain (ADG), fecal score in pens and serum and tissue zinc (Zn) levels around time of weaning in order to reduce the environmental impact without loss of the beneficial effect of ZnO on intestinal health and growth. Five case herds with enterotoxic colibacillosis challenges were included. The piglets entered the study aged three or five weeks. All piglets received a commercial diet containing <150 mg Zn/ per kg of complete feed. Four treatment groups received commercial peat added A: uncoated ZnO, B: lipid microencapsulated ZnO, C: solely commercial peat or D: no peat (Farms 2 and 3).

**Results:**

At Farms 1, 2 and 3, a significant effect of treatment was identified for fecal score (*P* < 0.05). Treatment A led to lower fecal scores compared to treatments C (*P* < 0.05) and D (*P* < 0.01). At Farms 2 and 3, there was a significant difference in individual average daily weight gain (iADG) between treatment A and D (*P* < 0.05). The iADG of piglets receiving treatment B did not differ significantly from treatment A.

**Conclusions:**

In 2016, The European Medicines Agency’s Committee on Veterinary Medicinal Products concluded that the benefits of ZnO for the prevention of diarrhea in pigs do not outweigh the risks to the environment. Effective alternative measures to reduce the accumulation of Zn in the environment have not been identified. Our results imply that peat added low concentration of both coated and uncoated ZnO influences the gut health of weaned piglets reflected by enhanced weight gain and reduced occurrence of diarrhea. This preventive approach certainly represents a favourable alternative in the “One Health” perspective. It will also contribute to reduced antibiotic use in pig farming while diminishing the environmental consequences caused by ZnO.

## Background

Piglets are vulnerable to *Escherichia coli* (*E. coli)* infections around weaning since circulating plasma antibodies are low and passive intestinal immunity provided by antibodies in the sow’s milk (IgA) is lost when the sow and piglets are separated [1, 2]. The separation from the sow, a different environment, commingling with unfamiliar piglets, hierarchy establishment through fighting and abrupt change in nutrition, are major changes that show a negative influence on immune functions and may result in *E. coli* associated post-weaning diarrhea (PWD) or edema disease (ED) [3–7].

*E. coli* causing PWD or ED enter the organism by ingestion and colonize the small intestine after attaching to porcine receptors on the enterocytes with fimbrial adhesins. The degree of colonization determines whether clinical manifestations occur or not. Fimbriae-designated *E. coli* F18^+^ and F4^+^ are typical pathogens involed in ED and PWD, *E. coli* F5^+^, F6^+^ and F41^+^ can occur in suckling pigs [8, 9]. Enterotoxigenic *E. coli* (ETEC) strains cause watery diarrhea and systemic disease in piglets due to their ability to colonize the intestine through expression of adhesins and the ability to produce the toxins heat labile (LT) or heat stable (ST) enterotoxins as well as Shigatoxin 2e (STx2e) [10–12]. The ED causing hemolytic *E. coli* F18ab^+^ produce Stx2e [13].

Since 2006, the use of antibiotic growth promoters in piglets has been banned in Europe [14]. As a consequence, there has been an increase in some uses of ‘therapeutic’ antibiotics and possibly switches to different and more modern antibiotics [15]. Therefore, AR development in production animals remain a serious problem in consumer health protection. Various natural materials such e.g. organic acids or zinc (Zn) have been tested as alternatives to antibiotics.

Zinc oxide (ZnO) at therapeutic concentrations (2000 ppm or more) has been widely used to prevent porcine colibacillosis, improve suboptimal weight gain and feed efficiency. The small-intestinal mucosa is altered by feeding of 3000 ppm Zn for 14 days [16], thus potentially increasing the absorptive capacity of the small intestine and consequently improving growth. Dietary treatment with ZnO has been associated with significant differences in the transcript abundance of several genes. Dietary ZnO supplementation influence metallothionein mRNA expression in the intestine and liver, enhances expression of the tight junction genes occludin and ZO-1 both at mRNA and protein levels and further enhances small intestinal IGF-I and IGF-I receptor gene expression, which can explain improved intestinal health [16–18]. An influence on the gastrointestinal microbiota in weaned piglets has been described [19–21]. Reduced fermentation of digestible nutrients in the proximal part of the gastrointestinal tract may render more available energy for the host and contribute to the growth-promoting effect of high dietary ZnO doses [20].

ZnO tend to dissociate after uptake in the low pH environment of the anterior gastrointestinal tract, allowing interaction with other nutrient and ingredient potentially leading to impaired absorption, and thus decreased bioavailability [22]. Long term use of pharmacological ZnO concentrations (2000 to 4000 mg Zn/ kg) to pigs feed, increases the concentration of Zn in the pig manure and results in Zn accumulation of arable land [23, 24]. Accumulation of Zn in soils may impose a toxicity risk on plants and micro-organisms [25]. Therefore, European feed legislation limits total dietary Zn in complete feed to 150 ppm [26]. Recent studies also support the assertion that Zn in feed may favour or select for AR [27–32].

Microencapsulated ZnO products are available on today’s market. According to the manufacturers, lipid microencapsulation avoids ionization of the active component in the animal stomach. Therefore biological properties are preserved and the biological effects are excerted in the animals’ small intestine [33, 34]. Studies have stated that coated ZnO at inclusion levels between 100 and 200 ppm in basal diets show similar effects as uncoated ZnO on growth promotion, reduced incidence of diarrhea and microbiota composition regulation [33–36].

A different approach to prevent intestinal disease in weaned piglets is peat supplementation, which has been associated with beneficial effects on health status, growth and mortality rates [37–39]. Peat contain humic substances including humic acids showing detoxifying properties because of chelate formation with potentially toxic substances such as heavy metals [40, 41], stimulation of digestion [42] and anti-inflammatory and antiviral effects [43, 44]. Due to its low pH (3.0 to 5.5) peat causes a reduction of the pH of the intestinal contents with subsequent reduced growth of *Enterobacteriaceae* [38]. Humic and fulvic acids have shown to improve nutrient uptake in suckling piglets [45]. Limited literature is available regarding the optimal dosage of peat preparations to piglets. An inclusion level of 0,5% humic substances to dietary treatments may improve ADG in weaned piglets [46]. Independent studies have stated that the effects of batches containing dietary humic substances are variable, warranting further investigation [39, 46]. An important prerequisite when applying peat supplementation to animals, is the monitoring of potentially pathogenic mycobacteria sometimes present in batches of peat preparations [47, 48]. These case herd trials aimed to explore the effects of different ZnO sources and ZnO levels in peat preparations on pre- and post-weaning fecal consistency and weight gain. Additionally, initial and final Zn-serum concentrations, liver and kidney Zn levels were examined at two of the farms.

A feed additive consisting of peat and uncoated or coated ZnO was produced. We anticipated that this preparation would show both peat and ZnO derived beneficial effects and therefore low ZnO levels added to peat should show the same effects as pharmacological ZnO levels added to feed. We hypothesized that low level ZnO enriched peat would improve piglet average daily weight gain (ADG) and reduce fecal score.

## Methods

### Preparation and quality control of Zn oxide enriched peat

Commercial peat (Pluss Avvenningstorv, Felleskjøpet, Norway) was used as the peat substrate in treatments A, B and C. It contained 31.1% dry matter and <5 mg Zn/ kg at a pH of 5.3. The contents of molds and yeasts were below 100 CFU/ g, no *Salmonella spp*. were detected and bacterial colony counts (30 °C) were 9000 CFU/ g. The commercial peat was analyzed by Alcontrol Laboratories (Stjørdal, Norway) by employing standard methods (Dir152/2009/EU [49], ISO 6869 [50], NMKL 98 [51], NordVal no: 014 [52], Tecra [53], NS-EN ISO 10523:2012 [54]).

Zn sources used to prepare the peat supplements included uncoated Normin Sink®, (8% Zn, Normin, Hønefoss, Norway) and lipid microencapsulated Zincoret® S (0.3% Zn, Vetagro S.p.A, Reggio Emilia, Italy). ZnO enriched peat was prepared by Fossli AS (Frosta, Norway). Peat A was commercial peat added 2819 mg/L uncoated ZnO (2255 mg Zn/L). Peat B was commercial peat added 321 mg/L coated ZnO (257 mg Zn/L). Peat C was commercial peat without ZnO-additives. Uncoated or coated ZnO was added to a batch of peat and transported in sacks of 80 L. The farmers and veterinarians (authors) were double-blinded to the composition of the treatment groups. The Zn content in peat preparations was analyzed prior to the start of the trial by standard methods at an accredited laboratory (Labnett Laboratories, Stjørdal, Norway) [55]. Peat was analyzed at the Norwegian Veterinary Institute in Oslo by a polymerase chain reaction to identify strains of *Mycobacterium avium* with specific primers (IS901-IS902) [56, 57]. No pathogenic *Mycobacteria avium* were detected by applying IS901-IS902 specific primers on samples isolated from the peat preparation.

### Case herds

Five Norwegian commercial pig herds with a documented history of clinical disease associated with *E. coli* infections were recruited (Table 1). Fecal samples from the case herds were submitted to serological testing (agglutination in microtiterplates with boiled antigen and single O-antisera) or PCR analyses conducted at the Norwegian Veterinary Institute or at the Norwegian School of Veterinary Science, respectively. At Farm 1, diarrhea occurred repeatedly around 3 weeks of age. At Farms 2–4, PWD was observed regularly. At Farm 5, ED had caused significant losses across multiple batches.

**Table 1:** Documented virotypes and serotypes of *E. coli* causing ETEC and STEC at the case herds 1 to 5 prior to performing trials

Farm	Piglet genotype	Pathotype	Virotype	O serogroups
1	LYLL	ETEC	F4	
2	LYHH	ETEC		O149
3	LYLL	ETEC		O138
4	LYLD	ETEC	LT:STb	
5	LYHH	STEC	Stx2e:F18	O139

The pathotypes, virotypes and O serogroups verified at the case herds 1 to 5 prior to recruitment for the trials are listed. Strains of *E.coli* isolated from fecal samples of piglets after post mortem examination were either forwarded by the veterinarian in charge to the Norwegian Veterinary Institute for serotype determination (agglutination in microtiterplates with boiled antigen and single O-antisera) or to the Norwegian School of Veterinary Science in Oslo for virulence pattern determination (PCR analysis). The pathotype Enterotoxigenic *E. coli* (ETEC) was found at Farms 1 to 4 while Shiga-toxigenic *E. coli* (STEC) was found at Farm 5. Virotypes at Farms 1, 4 and 5 were F4, LT:STb and Stx2e:F18, respectively. The O serogroups O149, O138 and O139 were identified at Farms 2, 3 and 5, respectively. Genetic combinations of piglets included Landrace x Yorkshire (LY) x Landrace x Landrace (LL) (LYLL) at Farm 1. At Farm 2, LY x Hampshire/Hampshire (HH) (LYHH) were used. At Farm 3, LYLL piglets were utilized. LY x Landrace/Duroc (LYLD) and LYHH were used at Farms 4 to 5, respectively

All dams were vaccinated against *E. coli* (recombinant adhesin F4 (F4ab, F4 ac, F4ad), recombinant adhesin F5, field strain adhesins F6 and F41) for passive transfer of lactogenic immunity in the suckling piglets.

### Animal management and measurements

The trials began with piglets aged two weeks on Farm 1 whilst on Farms 2–5, piglets close to five weeks (the average weaning age in Norway) of age were enrolled in the trials. Across all farms, piglets (females and castrated males) were weaned at 32 to 33 days of age (10.16 ± 1.80 kg of bodyweight (BW)). Digital thermometers were used to monitor the room temperatures at Farms 1 to 3. At Farm 1, room temperature was 18 °C ± 2 °C and the temperature on the piglet creep floor, measured with a handheld laser device, was 24–25 °C. At Farms 2 and 3, room temperature showed fluctuations (day and night) between 23 and 21 °C at initiation of the trials. The room temperature was gradually reduced (0.5 °C per day) and set to 18 °C. At Farms 4 and 5, the temperature was set to 22 °C at initiation of the trials and reduced gradually to 18 °C. The light (L): dark (D) periods were 16 L:8D. At Farm 1, the piglets were fed their basic feed on the floor. Restrictive feeding was conducted at Farms 2 and 3 while Farms 4 and 5 practiced ad libitum feeding. The pigs had free access to drinking water. Natural wood shawings were used as bedding material at all farms.

Concentrate feed used at all five trial farms was a standard starter feed (Table 2). Pens of 10 to 12 piglets were allocated to one of three treatments (A to D). A farmers consent to include a control group (treatment D) was attained at Farms 2 and 3.

**Table 2:** Composition of basal feed at trial farms

Farm:	Farm 1	Farm 2	Farm 3	Farm 4	Farm 5
Production stage:	PrW^a^	PoW^b^	PoW	PoW	PoW
	Feed composition				
Crude protein (%)	16.00	19.60	18.30	18.00	18.10
Dry matter (%)	88.00	88.20	86.90	87.40	88.00
Lysine (%)	1.23	1.39	1.20	1.20	1.28
Crude fat (%)	3.00	6.00	5.10	5.30	4.90
Crude ash (%)	5.00	4.70	5.00	4.50	5.20
Vit. A (IU)	10,000	8000	10,000	8000	10,000
Vit. D (IU)	1500	1300	1000	1500	1500
Vit. E (mg)	200	180	150	150	200
Copper sulphate (mg/kg)	15	26	15	32	15
Sodium selenite (mg/kg)	0.20	0.10	0.30	0.40	0.20
6-phytase (FYT/kg)	500	500	1500	703	500
Zn (mg/kg)	120	141	120	141	120

Composition of basal feed fed at the five trial farms. The levels of crude protein varied between 16% (Farm 1, preweaning phase) and 19.6% (Farm 2, postweaning phase). Vitamin levels were comparable. Sodium selenite levels varied between 0.1 and 0.4 mg/kg

^a^PrW: Pre-weaning

^b^PoW: Post-weaning

The treatment duration at each case farm was decided based on known challenges with *E. coli* infections and the expected duration of clinical cases based on the farmers previous experiences. *E. coli* associated diarrhea in suckling piglets from 2 to 4 weeks of age was a documented herd health problem at Farm 1. Therefore, treatments A to C were provided from 2 weeks of age until weaning. One liter of peat A, B or C was provided to each pen twice a day. At Farms 2, 3 and 4, experiencing repeated cases of *E. coli* associated PWD, treatments with 1 L peat/ pen twice a day were initiated at weaning (day 0) and continued for 14 to 17 days.

Farm 5 struggled with repeated cases of *E. coli* associated ED. Due to the known presence of a highly ‘aggressive’ *E. coli* strain, animals received 2 L daily of treatments A, B or C starting one week before weaning (day −7). The next 2 weeks, the animals received twice the amount of peat compared to Farms 1, 2 and 3; 4 L daily of treatments A, B or C (day 0–14). The last week, these animals again received 2 L daily of treatments A, B or C (day 15 to 21 after weaning) as the abrupt withdrawal of ZnO supplementation may favour the growth of Shiga-toxigenic *E. coli* (STEC).

BW was registered and blood samples were collected from randomly selected and ear tagged piglets at Farms 2 (*n* = 12) and 3 (*n* = 6) via the external jugular vein prior to study entry at weaning (day 0). The same piglets were bled and weighed individually at termination of the trials.

On Farm 1, all piglets in one farrowing unit were ear tagged and the body weight was registered on study entry, at two weeks of age (− day 21, three weeks before weaning) and day 0 (weaning day). On Farms 2 to 5, group weights of pigs within the same pens were recorded on day 0 and on the last day of the trial (day 14–21). Each trial pen was evaluated for clinical signs of disease (depression, signs of dehydration and perineal staining) and fecal consistency scores by the same veterinarian at each farm. Clinical signs of disease were not scored. A standardized four-point categorical fecal scoring system was used (score 1: firm, 2: pasty, 3: loose and 4: liquid feces). Observation of a pen with liquid feces (category 4) was scored as diarrhea, irrespective of the number of piglets affected. Rectal swabs on charcoal transport medium were taken from all piglets with fecal score 4 for bacteriological culture. No antibiotic treatment was applied at the case farms during the trials.

On Farm 2, three animals (*n* = 3) from each group A, B, C and D were euthanized by captive bolt gun and exsanguination on days 7 and 15 of the trial for collection of totally 24 fresh liver and 24 kidney samples.

### Serum and tissue Zn analysis

Blood samples were collected in 9 ml serum collection tubes coated with clot activator (Vacuette®, Med-Kjemi AS, Norway). All samples were directly transported to the laboratory within a maximum of 120 min following sample collection without any prior chilling. Samples were analysed to determine the following parameters: iron (Fe), inorganic phosphate (P), copper (Cu), zinc (Zn), calcium (Ca), magnesium (Mg) and ceruloplasmin (Cp) levels. Levels of Fe and P were assessed by a colorimetric method (ABX Pentra 400 Analyzer, Horiba). Cu, Zn, Ca, Mg and Cp were determined by Atomic absorption spectrometry (AAnalyst 300 Perkin Elmer). Due to limited financial resources, blood sampling was restricted to Farms 2 and 3.

Tissue samples were stored at −20 °C until analysis. Inductively coupled plasma mass spectrometry (ICP-MS) was performed by SYNLAB.vet GmbH (Berlin, Germany) to determine Zn concentrations in liver and kidney samples.

### RNA extraction and reverse transcription

A multiplex PCR analysis was conducted on *E. coli* isolates from affected piglets at the five farms included in this study. Total RNA from bacterial pellets was extracted using the RNAeasy Mini Kit (Qiagen, Hilden, Germany) according to the manufacturer’s instructions (Protocol 4 and 7) including an on-column DNA wipeout treatment (Appendix B1–4). The RNA was eluted in 30 μl DEPC-treated water (Invitrogen) and stored at −70 °C until reverse transcription (RT). Gel electrophoresis with 1% agarose gel was used to confirm that isolated RNA was intact while the concentration and purity of the RNA extracts were analyzed by measuring the absorbances at 260 (A260) and 280 nm (A280) using a NanoDrop™ ND-1000 spectrophotometer (Thermo Scientific, Waltham, MA, USA). Only total RNA samples of high quality with A260/A280 ratios between 1.9 and 2.2 and with tight bands of 18S/28S ribosomal RNA (rRNA) were used for RT.

Reverse transcription was conducted with QuantiTect® RT kit (Qiagen) according to manufacturer’s instructions for the synthesis of complementary DNA (cDNA) and included a DNase wipeout treatment. Amounts of 1 μg of RNA were used in each RT reaction conducted in a BioRad T100 (Bio-Rad, Hercules, CA, USA). In addition, to confirm the absence of any contamination with genomic DNA (gDNA) contamination, one RNA sample per round of extraction was randomly chosen and not treated with reverse transcriptase. The cDNA samples were diluted in 180 μl of DEPC-treated water and stored at −70 °C until.

### Multiplex polymerase chain reaction (PCR) analysis

The *E*. *coli* strains isolated at the five case farms were characterized by applying primer sequences obtained from previous publications, targeting for the following genes/ virulence factors: (a) estB/ STb [58], (b) estA/ STa [59], (c) eltB/ LT [60], Stx2e (A subunit) [61], faeG/ F4 [62], fanA/ F5 [63], fasA/ F6 [64], fedA/ F18 [65] and fedA subunit/ F41 [66].

### Statistical analyses

Data maintained on individual animal and pen-level measurements were managed in Excel (Microsoft, Windows). Data analyses were performed in STATA version 13.1 (StataCorp LP, College Station, TX) and JMP® Pro version 12.1.0 (Cary, NC, USA). Descriptive statistics and graphical plots were used to assess for any visual differences in the population starting weight, mean and range of fecal scores.

Individual piglet weight data collected at trial entry and completion was used to derive three outcome variables. The individual daily weight gain (iADG, g/ day) was calculated as overall weight gain/days in study. Then, a group-level outcome was calculated; the average daily weight gain (ADG) was calculated in gram per day (g/ day) as mean weight gain of each treatment group/ days in study. Duration variation was corrected for in statistical analyses.

To investigate whether there was a significant effect of treatment type on iADG and ADG, mixed effects linear regression models were fitted in Stata version 13.1 (Statacorp, TX). Treatment type was included as a fixed effect, and farm identity (1 to 5) as a random effect. The effect of gender (female or castrated male), pen identity and PDI were also examined as fixed effects in separate univariate models.

Mixed effects regression models were also used to examine the effect of treatment type (A to D) on fecal score, serum and tissue mineral levels in separate univariate models. Likelihood ratio tests were used to assess model significance. Model outcomes were described using coefficient β (indicating the magnitude of the effect), the 95% confidence interval (CI) and Wald *p*-values [67]. To assess the effect of treatment type, the baseline (β = 0) for comparison of coefficient values was set as treatment A (peat containing 2819 mg/L uncoated ZnO).

## Results

### Peat preparation quality

No pathogenic *Mycobacteria avium* were detected by applying IS901-IS902 specific primers on samples isolated from the peat preparation.

### Fecal scores

Regression models found no effect of farm identity or pen identity or fecal score (*P* > 0.05). However, treatment type had a significant effect on fecal score (*P* < 0.05). Compared to pens of piglets receiving treatment A (β = 0), higher fecal scores, indicative of looser feces, were recorded in pens receiving lower levels of Zn inclusion - treatments C or D (*P* < 0.05).

### Pen average daily weight gain (ADG) and individual daily weight gain (iADG)

Outbreaks of PWD and ED on Farms 4 and 5 caused mortality rates of 2.9% and 6.2%, respectively. Due to high mortality and reduced weight gain observed in affected piglets, data from Farms 4 and 5 were analyzed separately to look at the effects of different ZnO-treatments on weight gain. A summary of weight gain results are listed in Table 4.

On farm 1, no significant effect of treatment was found for iADG. Gender did not have a significant effect on iADG.

Mixed-effects models identified significantly lower iADG (*P* < 0.05) in piglets receiving treatment D, comparing with those receiving treatment A at Farms 2 and 3 (Table 4). At Farm 2, iADG in treatment groups A, B, C and D were 410 ± 90 g/ day, 390 ± 100 g/ day, 340 ± 150 g/ day and 290 ± 130 g/ day.

At Farm 3, iADG in treatment groups A, B, C and D were 410 ± 110 g/ day, 370 ± 100 kg/ day, 270 ± 80 g/ day and 230 ± 80 g/ day.

### Effects on serum minerals and tissue Zn concentrations

Data from Farms 2 and 3, indicated that serum Fe, P, Cu, Ca and Mg levels were not influenced by treatment. Linear regression analysis suggested that treatment had an influence on serum Zn serum levels at Farm 2. Compared to treatments B-D, treatment A was associated with a significantly higher Zn concentration increase in serum after 14 days of treatment (*p* < 0.02). Mean serum Zn (SD) increase was 3.43 (2.42) μmol/ L and 1.71 (2.25) μmol/ L for treatments A and B, respectively. Treatment C lead to an increase of 0.27 (2.32) μmol serum Zn/ L and treatment D lead to an increase of 1.03 (2.99) μmol serum Zn/ L. At Farm 3, no significant serum Zn increase was observed, when comparing initial and final Zn serum levels across all treatment groups.

Mean Zn concentrations (μg/ g dry weight) in liver samples of piglets at Farm 2 receiving treatments A to D for 7 days were 31.9 (4.3), 25.5 (3.1), 20.9 (2.5) and 25.7 (1.8), respectively. Mean Zn concentrations (μg/ g dry weight) in liver samples of piglets at Farm 2 receiving treatments A to D for 15 days were 57.8 (10.42), 51.37 (7.05), 29.30 (7.78) and 25.77 (3.98), respectively. Mean Zn concentrations (μg/ g dry weight) in kidneys of piglets at Farm 2 receiving treatments A to D for 7 days were 13.5 (0.9), 13.5 (0.8), 13.5 (0.2) and 12.3 (0.5), respectively. Mean Zn concentrations (μg/ g dry weight) in kidneys of piglets at Farm 2 receiving treatments A to D for 15 days were 30.23 (6.91), 27.23 (9.42), 11.95 (4.03) and 11.06 (2.48), respectively.

### Clinical signs and bacteriology

Bacteriological investigations of fecal material from piglets observed with clinical signs of diarrhea revealed that pathogenic *E. coli* strains were isolated from all five farms (Table 3). At Farm 1, ETEC and the two predominant virotypes STa:F5:F41 and LT:STb:F4 were isolated. Signs of diarrhea were evident across treatment groups at initiation of the trial. Only one pen in treatment group C showed signs of diarrhea until eight days into the trial. ETEC STb^+^ were the predominant pathotype and virotype at Farm 2 and clinical signs of diarrhea were seen at initiation of the trial in pens across treatment groups. The symptoms disappeared in all treatment groups except the group receiving no peat. At Farm 3, ETEC F18^+^ was found and clinical symptoms occurred seven days into the trial in pens where piglets received treatment C and D. At Farm 4, ETEC LT:STb:F4^+^ was associated with an outbreak of sudden death affecting 16 piglets (2.9% of the batch) within a one week period, starting 3 days prior to weaning. Post-mortem examination revealed hemorrhagic enteritis in all examined piglets. At Farm 5, STEC Stx2e:F18^+^ caused sudden death of totally 24 piglets (6.2% of the batch) within a two weeks period, the first cases occurred at weaning. Post-mortem examinations of several piglets revealed macroscopic pathological findings compatible with ED including subcutaneous edema, edema in the submucosa of the stomach and the mesocolon. One trial piglet in the control group (D) at Farm 3 died during the experimental period. Necropsy findings were consistent with a case of haemorrhagic enteritis caused by *E. coli* infection. Serotyping and multiplex PCR analysis revealed an *E*. *coli* 0138 F18^+^strain.

**Table 3:** Pathotyping and virotyping of *E. coli *strains isolated during trials at Farms 1 to 5

Farm	Pathotype	Virotype	Fimbriae and toxin prevalence (%)
1	ETEC	STa:F5:F41	50.0
		LT:STb	12.5
		LT:STb:F4	37.5
2	ETEC	STa:STb	13.3
		STa:STb:F4	13.3
		STxA	13.3
		STb	46.7
		STb:F18	6.7
		LT:STb	6.7
3	ETEC	F18	100.0
4	ETEC	LT:STb:F4	100.0
5	STEC	Stx2e:F18	100.0

*E. coli* isolates from Farms 1 to 5. Pathotypes and virotypes are described. At Farms 1 to 4, the pathotype Enterotoxigenic *E. coli* (ETEC) was found. At Farm 5, Shiga-toxigenic *E. coli* (STEC) was present. At Farms 1 to 5, the predominant virotypes detected by multiplex PCR analysis were STa:F5:F41, STb, F18, LT:STb:F4 and Stx2e:F18, respectively

## Discussion

Therapeutic ZnO levels in diets for weaner pigs to prevent *E. coli* infections are widely used as an efficient and cost-effective preventive strategy for PWD or ED [68–70]. In Asia and the Americas, it has been standard procedure to apply up to 3000 ppm of ZnO in weaners feed [71]. However, various studies have elucidated different challenges associated with this prophylactic approach including antimicrobial resistance and environmental pollution [25, 28, 29, 72–74]. This study aimed to identify whether peat supplemented with low-level uncoated or coated ZnO preparations could offer a feasible and effective alternative to conventional therapeutic ZnO levels for the reduction of *E. coli* associated diarrhea or ED. To the authors’ knowledge, there are no previous reports on the effects of coated ZnO enriched peat on weaned piglets production parameters. Data from the present study supported our hypothesis that feeding ZnO enriched coated peat to weaned piglets for 14 days can achieve the combined beneficial effects of higher weight gain and reduced fecal scores. Additionally, the usage of coated ZnO to prevent enterotoxic colibacillosis can reduce Zn emmissions from swine producing units resulting in a substantially lower environmental impact.

An effect of treatment type on fecal consistency scores was found at Farms 1 to 3. Treatment C (commercial peat without ZnO-additives) and treatment D (controls) resulted in significantly higher fecal scores than treatment A (2819 mg/L uncoated ZnO) at Farms 1 to 3. These findings are consistent with a previously published study showing that 14 days of post weaning ZnO-inclusion in feed affected fecal consistency, and that 3125 mg/kg of uncoated ZnO led to firmer fecal consistency than the inclusion of 139 mg/kg feed of a lipid encapsulated ZnO source called Shield Zn [75]. In the present study, basal diets at Farms 1–3 contained between 16% and 19.6% crude protein. Farms feeding diets containing crude protein levels below 19% can be considered relatively low. Low-protein diets may have a diarrhea-reducing effect [76]. This, combined with a possibly suboptimal concentration of ZINCORET™ included in treatment B (321 mg/L coated ZnO), may have concealed the presumptive effect on fecal consistency. Thus, any future studies including coated ZnO in peat may require concentrations above 321 mg ZnO/ l to promote significant effects on fecal consistency and growth rates. Other reports have described beneficial effects of coated ZnO on growth rates, intestinal morphology, digestive enzyme activity and colibacillosis at rates of 100 to 200 ppm in basic feed to recently weaned piglets [28, 75, 77].

ZnO treatment had no significant effect on ADG on Farms 1, 2 and 3. Large body weight variations within each group combined with a relatively low number of pens per treatment may have contributed to these results. No treatment effect on iADG was discovered at Farm 1. These piglets entered the study at three weeks of age. A possible explanation to the lack of statistical differences in weight gain across the groups at Farm 1, may be related to the fact that the farmer did not consent to include a control or untreated group (treatment D) due to concerns regarding *E. coli* associated diarrhea. At Farms 2 and 3, however, treatment D was included. Ear tagged piglets receiving treatments A showed higher iADG than piglets receiving treatment D. Individual animal identification through ear tagging and individual weight measurements of a larger number of piglets across the study farms could have provided stronger evidence to support the finding of growth promoting effects of ZnO enriched peat treatment identified on Farms 2 and 3.

Serum Zn serum levels at Farm 2 were influenced by the type of Zn treatment. Compared to treatment A (2819 mg/L uncoated ZnO), treatment B (321 mg/L coated ZnO), C (commercial peat without additives) and D (controls) were associated with significantly lower increases in serum Zn concentration after 14 days of treatment. Our results are consistent with previous studies, showing that inclusion of ZnO in the feed will increase the serum Zn concentrations [78–80].

Tissue samples from only three animals per treatment group were collected at days 7 and 15 of the first trial at Farm 2 due to limited financial resources. Although no statistically significant effects of treatment were seen on final Zn kidney and liver concentrations, the highest numerical increase of both liver and kidney Zn concentrations was observed in animals receiving treatment A, followed by animals receiving treatments B, C and D, respectively.

These results are in line with previous findings, showing greater hepatic and circulating Zn concentrations in piglets receiving therapeutic concentrations of uncoated ZnO (between 2000 ppm and 2500 ppm) than piglets fed 100 to 200 ppm of coated ZnO [28, 29, 81]. The mean Zn concentrations in liver samples from piglets receiving treatment A were 57.80 (10.42) μg/ g dry weight while Zn concentrations in liver samples from piglets receiving treatment B, C and D were 51.37 (7.05) μg/ g dry weight, 29.30 (7.78) μg/ g dry weight and 25.77 (3.98) μg/ g dry weight, respectively.

Peat B contained 321 mg/L coated ZnO while Peat A contained 2819 mg/L uncoated ZnO, equivalent with a ratio of 1:8.8. Despite the low coated versus high uncoated ZnO ratio in Peat B and Peat A, comparable Zn-levels were detected in animals receiving Peat A or Peat B. These tissue Zn concentrations suggest that the bioavailability of coated Zn added to peat is higher than uncoated Zn. Experimental studies on the pharmacological effects of Zn to reduce post-weaning scouring and improve body weight gain have shown that formulations of Zn in organic form or lipid-encapsulated Zn may be effective at relatively low concentrations, achieving comparable effect with far higher concentrations of inorganic Zn. This indicates that the bioavailability and retention of organic form or lipid-encapsulated Zn may be increased [24]. A recent study demonstrates that nanosize ZnO can increase Zn digestibility, serum growth hormone levels and carbonic anhydrase activity and enhance the immune response of weanling piglets [82]. Uncoated ZnO tend to dissociate after uptake in the low pH environment of the anterior gastrointestinal tract, allowing interaction with other nutrient and ingredient potentially leading to impaired absorption, and thus decreased bioavailability [22].

The fecal Zn concentrations were not measured in this trial due to limited financial resources, but it seems likely that fecal Zn concentrations in feces from piglets receiving treatment B would be lower than Zn concentrations in feces from piglets receiving treatment A, as shown in previous studies [83–85].

Five farms with a history of *E. coli* associated enteric disease were specifically included in this study. Pathogenic *E. coli* strains were detected at all farms. During the course of the trial, clinical signs of disease were not evident on Farms 1 and 2. At Farm 3, one piglet died due to haemorrhagic enteritis associated with *E. coli* whilst Farms 4 and 5 experienced outbreaks of *E. coli*-associated peracute-to-acute PWD and ED, respectively. The differences in clinical presentations may be explained by the fact that different pathotypes of *E. coli* were present at the different trial farms (Table 3). Possible coinfections with e.g. rotavirus [86], management, feeding and hygiene policies [87–89] may also have influenced the general enteric health and disease susceptibility among piglets at Farms 4 and 5. The detection of coinfection-causing agents was not included in this study. The passive protection of piglets against *E. coli* infections through vaccination of the dams decreases with ageing and lactogenic immunity suddenly stops at weaning [90]. It is likely that subclinical infections of the surviving weaned piglets at Farms 4 and 5 affected ADG. Reduced weight gain is indeed associated with subclinical infections in pigs [91–93]. Tables 4 shows that the ADG/ piglet in treatment groups A to C were low at Farms 4 and 5 where ETEC and STEC expressing the virulence factors LT:STb:F4 and Stx2e:F18 were found. Mortality rates of 2.9% at Farm 4 and 6.2% at Farm 5 during the first 2 weeks after weaning occurred in spite of prophylactic treatment with ZnO enriched peat. This may suggest that both uncoated or microencapsulated ZnO-concentrations in peat require optimization to achieve broader preventive effects on piglets at farms with different infection pressure and *E. coli* variants.

**Table 4:** Average daily gain (ADG) results at Farms 1 to 5

Farm	Treatment	Trial duration (days)	ADG (g/ day)	SEM	n=	iADG (g/ day)	SEM	n=
1	A	21	270	12	6	270	12	57
	B		300	11	6	300	11	54
	C		280	12	6	280	12	57
2	A	15/15	400	12	9	410^a^	18	12
	B		410	11	9	390	21	12
	C		340	10	9	340	31	12
	D		330	12	9	290^b^	27	12
3	A	15/17	400	5	6	410^a^	31	6
	B		370	10	6	370	29	6
	C		320	8	6	270	23	6
	D		370	8	6	230^b^	23	6
4	A	15	220	3	18	#	#	#
	B		210	2	18	#	#	#
	C		190	4	17	#	#	#
5	A	14/15	230	8	12	#	#	#
	B		240	8	12	#	#	#
	C		210	7	12	#	#	#

The pen based and individual weight measurements are expressed as ADG/ piglet and iADG, respectively. Treatment A was peat containing 2819 mg/L uncoated ZnO (2255 mg Zn/L). Treatment B was peat containing 321 mg/L coated ZnO (257 mg Zn/L). Treatment C was commercial peat without ZnO-additives (36 mg Zn/L). Treatment D implied no feeding of peat (control groups). Individual measurements of all piglets were included at Farm 1, both group and selected individual weight registrations were included at Farms 2 and 3. Group weight registrations only were included at Farms 4 and 5. Significant differences (*P* < 0.05) between iADG at the Farms 2 and 3 are indicated by different superscripts (a or b). At Farm 3, two trials with differing duration were conducted without affecting the weight gain of piglets significantly (data not shown). Variation in trial durations were based on the need for farmers compliance to participate in the trials and the practicality at each farm. Duration variation was corrected for in statistical analyses

^#^Not included in the trial

The multiplex PCR results show that ETEC F4^+^ were found at both Farms 2 and 4. There was a clear difference in strain virulence at these farms, which may be explained by differing management strategies. At Farm 2 a strict cleaning and disinfection regime between batches was maintained. This was not possible at Farm 4 due to poor growth rates and consequently a reduced duration of empty periods between batches. Additionally, a large amount of flies were present at Farm 4. Flies are known to transmit bacteria including *E. coli* [94, 95].

The actual daily consumption of peat per piglet was not feasible to measure in this study because piglets were kept in groups. Instead, an estimated daily piglet Zn consumption rate was calculated, by dividing the amount of daily added Zn in peat preparations by the number of piglets per pen [96, 97]. This field trial demonstrated that significant growth promoting and diarrhea reducing effects were maintained by adding 2819 mg uncoated ZnO/ l of peat (Treatment A). Pens of piglets receiving 2 L of peat per day, received 5638 mg ZnO per day, which equals to 4510 mg Zn. Each piglet (10 to 12/ pen) should theoretically consume approximately between 451 mg Zn and 376 mg Zn per day. Treatments B equated to an approximate daily Zn uptake per piglet between 51 mg Zn per day and 43 mg Zn per day. Treatment C equated to an approximate daily Zn uptake per piglet between 7 mg Zn per day and 6 mg Zn per day. These levels of Zn uptake per piglet per day are much lower than the uptake levels when applying conventional pharmacological concentrations of ZnO in pelleted feed to piglets. If assuming a mean daily feed consumption of 450 g/ day during the first 14 days after weaning (32 days old piglets) [98, 99], piglets receiving conventional pharmacological levels of uncoated ZnO between 2000 and 3500 ppm (1600 and 2800 ppm Zn, respectively) added to their basal diet will consume between 720 and 1260 mg Zn per day, respectively. A comparison of feeding Peat A or Peat B with a diet added 2000 ppm uncoated ZnO (or 1600 ppm Zn) implicates a reduction of dietary Zn by 72.0% (Peat A) or even 96.8% (Peat B).

## Conclusions

To the author’s knowledge, this is the first field study undertaken to identify effects of supplementation with both uncoated and coated ZnO-enriched peat on fecal consistency and weight gain. This study has practical relevance for the control of enteric diseases in weaned piglets managed under European pig production systems. Since higher iADG was observed in a relatively small sample size of piglets receiving treatment A, our findings support the need for further research, conducted on a larger number of farms and under varying management conditions. The determination of optimal ZnO concentrations in peat preparations for growth-enhancing as well as PWD and ED preventive effects needs further investigation. Additionally, our promising findings support further investigation on coated ZnO in a larger randomized clinical trial, either added to peat or concentrates. Coated ZnO represents an alternative to reduce the negative impact on the environment and a way of counteracting potential co-selection for antibiotic resistance in bacteria.

In light of the current discussion regarding a possible ban on the use of ZnO in animal feed, it is important to emphasize that the usage of orally administered veterinary medicinal products containing ZnO should be reduced. Simultaneously, optimization of management strategies at pig producing units should always be strived for prior to applying ZnO as a preventive measure to avoid PWD or ED.

## Abbreviations

ADG: Average daily weight gain
AR: Antibiotic resistance
Ca: Calcium
CI: Confidence interval
Cp: Ceruloplasmin
Cu: Copper
*E. coli*: *Escherichia coli*
ED: Edema disease
ETEC: Enterotoxigenic *E. coli*
Fe: Iron
g: Gram
HH: Hampshire/Hampshire
iADG: Individual weight gain
L: Liter
LD: Landrace/Duroc
LL: Landrace x Landrace
LT: Heat-labile enterotoxin
LY: Landrace x Yorkshire
Mg: Magnesium
P: Inorganic phosphate
PCR: Polymerase chain reaction
PWD: Post-weaning diarrhea
Stb: Heat-stable enterotoxin B
STEC: Shiga-toxigenic *E. coli*
Zn: Zinc
